# Spatial-temporal distribution of genotyped tuberculosis cases in a county with active transmission

**DOI:** 10.1186/s12879-017-2480-z

**Published:** 2017-05-31

**Authors:** Saroochi Agarwal, Duc T. Nguyen, Larry D. Teeter, Edward A. Graviss

**Affiliations:** 1 Houston Methodist Research Institute, Houston, Texas USA; 2 Forensic Research and Analysis, 425 NW 10th Avenue, Ste 306, Portland, OR 97209 USA; 3 Institute of Academic Medicine, Department of Pathology and Genomic Medicine, Houston Methodist Research Institute, Mail Station: R6-414, 6670 Bertner Ave, Houston, TX 77030 USA

**Keywords:** *M. Tuberculosis*, Genotyping, Endemic *Mtb*, Clustering

## Abstract

**Background:**

Harris County, Texas is the third most populous county in the United States and consistently has tuberculosis rates above the national average. Understanding jurisdictional epidemiologic characteristics for the most common *Mycobacterium tuberculosis* genotyped clusters is needed for tuberculosis prevention programs. Our objective is to describe the demographic, laboratory, clinical, temporal and geospatial characteristics for the most common *Mycobacterium tuberculosis* GENType clusters in Harris County from 2009 to 2015.

**Methods:**

We analyzed data from the Centers for Disease Control and Prevention (CDC) Tuberculosis Genotyping Information Management System (TB GIMS). Chi-square analyses were used to determine associations between selected clusters and specific characteristics of interest. Geographical Information System (GIS) point density and hot spot maps were generated and analyzed with ArcGIS 10.4.

**Results:**

In Harris County from 2009 to 2015, 1655 of 1705 (97.1%) culture positive tuberculosis cases were genotyped and assigned a GENType, and 1058 different GENTypes were identified. The analyzed genotype clusters represent 14.1% (233/1655) of all genotyped cases: G00010 (*n* = 118), G00014 (*n* = 38), G00769 (*n* = 33), G01521 (*n* = 26), and G08964 (*n* = 18). Male gender (*p* = 0.002), ethnicity (*p* < 0.001), homelessness (*p* < 0.001), excessive alcohol use (*p* = 0.002), and U.S.-birth (*p* = 0.004) were associated with the 5 GENTypes. Hot and cold spots were identified as geographic areas having high and low TB incidence.

**Conclusions:**

Of more than 1000 distinct GENTypes identified in Harris County, there were 5 common *Mycobacterium tuberculosis* GENType clusters seen from 2009 to 2015. The common genotypes were observed primarily in U.S.-born populations despite the large foreign-born population residing in Harris County. GENType was significant distributed spatially and temporally in Harris County in the analyzed time period indicating that there may be outbreaks caused by transmission.

## Background

Genotyping of *Mycobacterium tuberculosis* (*Mtb*) isolates is used to identify tuberculosis (TB) cases that are closely related and when combined with epidemiologic data can be used to characterize jurisdictional *Mtb* strain transmission dynamics. The additional use of geospatial analysis can add another dimension to TB investigations and strengthen location-based surveillance.

Harris County (HC), Texas (TX), which includes the Houston metropolitan area, is the third most populous county in the United States (U.S.) and consistently has one of the highest TB rates in the U.S. (7.2 per 100,000 in 2015) [1]. To better plan and finance TB control and prevention strategies, responsible public health programs need an accurate description of current TB epidemiologic trends within jurisdictions including common genotype clusters and their epidemiologic characteristics.

In the current study, a cluster was defined as ≥2 *Mtb* isolates from different diagnosed TB cases having the same genotype, defined as the same spacer oligonucleotide genotype (spoligotype) and 24 loci variable number of tandem repeat – mycobacterial intersperse repetitive units (VNTR-MIRU) – GENType) occurring in a jurisdiction during a defined period [2]. Clustering is used as a surrogate marker for recent transmission unlike unique genotypes that are believed to indicate reactivation of a past infection [3] or recent migration of an infected TB case. In the U.S., 21.5% (4529/21,153) of genotyped TB cases were clustered between 2013 and 2015, and 30.5% (845/2742) were clustered in Texas during the same time period [2]. In Harris County the clustered proportion of genotyped TB cases from 2013 to 2015 was 45.0% (375/833). In the current study, we investigate and describe the 5 largest clusters of genotyped TB cases in HC between January 1, 2009 and December 31, 2015 and analyze epidemiologic trends of TB in this large, highly populated U.S. county.

## Methods

The Centers for Disease Control and Prevention (CDC) has a dynamic TB epidemiologic platform which stores information in the TB Genotyping Management System (TB GIMS) [4] including select patient-level surveillance data from the U.S. Public Health Service mandated (and required) Report of Verified Case of Tuberculosis [5] (RVCT) and genotyping cluster information such as GENTypes defined as *Mtb* isolates with identical spoligotype and VNTR-MIRU patterns. The CDC switched from using 12 loci to 24 loci VNTR-MIRU in 2009 to potentially increase the discriminatory power to identify *Mtb* clusters. Genotyped tuberculosis cases in TB GIMS from HC, TX between 2009 and 2015 were included in the analyses.

Chi-square analysis was used to determine if the given attribute differed by cluster by comparing various strata for the attribute among the 5 most common genotype clusters. All analyses were conducted in SAS 9.3 (Cary, NC). A *p-*value < 0.05 was considered statistically significant.

Epidemiologic curves were constructed for each of the analyzed clusters. TB case counts for each month based on count date from January 2009 through December 2015 were plotted for each individual GENType to visualize cluster dynamics over the study time period.

Geographical Information System (GIS) maps were created using ArcGIS 10.4 and 10.5 software (ESRI, Redlands, CA). Locations of TB cases were randomly placed in HC zip codes (based on an ArcGIS algorithm) where patients resided. A choropleth map displaying population estimates was created. Population data for each zip code was based on U.S. Census 2010 data [6]. Point Density maps were created using the Point Density Spatial Analyst tool. These maps present density as the number of cases per square mile. Hot spot analysis was conducted using the Optimized Hot spot Analysis option among the ArcGIS Spatial Analyst tools. Bilinear interpolation was used to smooth the contour look of the raster. Hot spot analysis utilizes a calculated z-score called a Getis-Ord Gi* statistic that is based on the intensity of a feature and the intensity of neighboring features. Larger z-scores indicate more intense clustering. To be a statistically significant hot spot a feature (zip code) has a local sum that is very different than expected local sum, so when that difference is too large to be the result of random chance the result is a significant z-score.

## Results

### Identification of clusters

In HC, 1655 of 1705 (97.1%) culture confirmed TB cases were genotyped and assigned a GENType between 2009 and 2015 among whom 45.7% (757/1655) were clustered. Five large genotyped clusters were identified in the specified time period. The analyzed large genotype clusters (5) represent 14.1% (233/1655) of all genotyped TB cases in HC: G00010 (*n* = 118), G00014 (*n* = 38), G00769 (*n* = 33), G01521 (*n* = 26), and G08964 (*n* = 18, Table 1). The 5 analyzed GENTypes were determined to be of either East Asian (L2) or EuroAmerican (L4) lineage (Table 1). All GENTypes with the East Asian lineage had the Beijing spoligotype “000000000003771” [7].

**Table 1:** Most Common GENTypes in Harris County, TX

Count	GENType	Spoligotype	MIRU	MIRU2	PCRType	Genotyping Lineage
118	G00010	000000000003771	223325173533	444534423428	PCR00002	East Asian (L2)
38	G00014	776037777760771	223125163324	242434223525	PCR00051	EuroAmerican (L4)
33	G00769	000000000003771	223325163333	444344223437	PCR00224	East Asian (L2)
26	G01521	000000000003771	223325173534	244544423239	PCR01201	East Asian (L2)
18	G08964	777703757760771	234325153321	441434223327	PCR04638	EuroAmerican (L4)

### Characteristics of clusters

Genotyped cases primarily occurred in men (74.3%, Table 2). GENTypes G00010 (61.9%) and G00769 (48.5%) occurred most often among Black/African Americans, but G00014 (52.6%), G01521 (42.3%) and G08964 (66.7%) occurred most often in individuals of Hispanic ethnicity (Table 2). Excessive alcohol use in the year preceding diagnosis was significantly different between the 5 GENTypes, and most common in GENTypes G01521 (76.9%) and G00014 (55.3%, Table 2). Based on birth country, 86.7% (202/233) of the TB cases among the 5 common GENTypes in HC occurred in U.S.-born residents. Of the 31 large-clustered cases in the foreign-born, 18 (58.1%) were in individuals born in Mexico. The remaining 13 cases were in individuals who originated from 7 countries in Asia and South America. The foreign-born had resided in the U.S. a median of 18 years before being diagnosed with TB disease (Table 2). Forty-five percent of the 5 large GENType cluster cases occurring in the foreign-born had GENType G00010.

**Table 2:** Characteristics of Cases Found in the Large Clusters in Harris County, TX

Variable	Total		G00010		G00014		G00769		G01521		G08964		*P*-value^a^
	*N* = 233	%	*n* = 118	50.64%	*n* = 38	16.31%	*n* = 33	14.16%	*n* = 26	11.16%	*n* = 18	7.73%	
Age													0.057
0–4	5	2.2	3	2.5	1	2.6	0	0.0	0	0.0	1	5.6	
5–15	2	0.9	0	0.0	0	0.0	1	3.0	0	0.0	1	5.6	
15–24	22	9.4	5	4.2	3	7.9	7	21.2	2	7.7	5	27.8	
25–44	64	27.5	35	29.7	9	23.7	8	24.2	7	26.9	5	27.8	
45–64	109	46.8	61	51.7	18	47.4	12	36.4	14	53.9	4	22.2	
65+	31	13.3	14	11.9	7	18.4	5	15.2	3	11.5	2	11.1	
Gender													0.002
Female	60	25.8	22	18.6	10	26.3	16	48.5	4	15.4	8	44.4	
Male	173	74.3	96	81.4	28	73.7	17	51.5	22	84.6	10	55.6	
Ethnicity													<0.001
White	42	18.0	18	15.3	4	10.5	10	30.3	8	30.8	2	11.1	
Black	112	48.1	73	61.9	13	34.2	16	48.5	7	26.9	3	16.7	
Asian	9	3.9	6	5.1	1	2.6	1	3.0	0	0.0	1	5.6	
Hispanic	69	29.6	21	17.8	20	52.6	5	15.2	11	42.3	12	66.7	
Other	1	0.4	0	0.0	0	0.0	1	3.0	0	0.0	0	0.0	
HIV status													0.252
Negative	176	75.5	86	72.9	27	71.1	28	84.8	22	84.6	13	72.2	
Positive	35	15.0	23	19.5	7	18.4	2	6.1	2	7.7	1	5.6	
Not Offered	22	9.4	9	7.6	4	10.5	3	9.1	2	7.7	4	22.2	
Homeless													<0.001
No	195	83.7	87	73.7	35	92.1	33	100.0	22	84.6	18	100.0	
Yes	38	16.3	31	26.3	3	7.9	0	0.0	4	15.4	0	0.0	
Excessive Alcohol													0.002
No	125	53.7	67	56.8	17	44.7	21	63.6	6	23.1	14	77.8	
Yes	108	46.4	51	43.2	21	55.3	12	36.4	20	76.9	4	22.2	
Drug Use													0.047
No	166	71.2	80	67.8	25	65.8	29	87.9	16	61.5	16	88.9	
Yes	67	28.8	38	32.2	13	34.2	4	12.1	10	38.5	2	11.1	
Origin									0	0.0			0.004
U.S. Born	202	86.7	104	88.1	29	76.3	31	93.9	26	100.0	12	66.7	
Foreign Born	31	13.3	14	11.9	9	23.7	2	6.1	0	0.0	6	33.3	
Country of Origin													<0.001
United States	202	86.7	104	88.1	29	76.3	31	93.9	26	100.0	12	66.7	
Mexico	18	7.7	8	6.8	5	13.2	0	0.0	0	0.0	5	27.8	
Vietnam	5	2.2	4	3.4	0	0.0	0	0.0	0	0.0	1	5.6	
Honduras	2	0.9	0	0.0	2	5.3	0	0.0	0	0.0	0	0.0	
Other	6	2.6	2	1.7	2	5.3	2	6.1	0	0.0	0	0.0	
Years in US median, (SD) [IQR]	18(13.2) [13,29]		22(15.1) [15,32]		17(9.3) [13, 17]		33.5(24.8) [16,51]		NA		18 (8.8) [12, 19]		0.490
Risk Factors													
Diabetes	36	15.5	16	13.6	7	18.4	7	21.2	4	15.4	2	11.1	0.795
Contact of infectious TB Pt	19	8.2	9	7.6	2	5.3	5	15.2	1	3.9	2	11.1	0.481
Post-organ transplant	1	0.4	1	0.9	0	0.0	0	0.0	0	0.0	0	0.0	0.913
End stage renal dis.	3	1.3	3	2.5	0	0.0	0	0.0	0	0.0	0	0.0	0.564
Immunosuppression	4	1.7	2	1.7	1	2.6	13	0.0	0	0.0	0	0.0	0.862
Clinical Characteristics													
Previous TB													0.706
No	219	94.0	110	93.2	35	92.1	32	97.0	24	92.3	18	100.0	
Yes	14	6.0	8	6.8	3	7.9	1	3.0	2	7.7	0	0.0	
SP smear													0.887
Negative	91	39.1	49	41.5	12	31.6	13	39.4	9	34.6	8	44.4	
Positive	120	51.5	58	49.2	23	60.5	17	51.5	15	57.7	7	39.9	
Not tested	22	9.4	11	9.3	3	7.9	3	9.1	2	7.7	3	16.7	
SP Culture													0.708
Negative	18	7.7	11	9.3	1	2.6	2	6.1	1	3.9	3	16.7	
Positive	193	82.8	94	79.7	34	89.5	28	84.9	23	88.5	14	77.8	
Not tested	22	9.4	13	11.0	3	7.9	3	9.1	2	7.7	1	5.6	
Chest X-Ray													0.518
Abnormal	213	91.4	104	88.1	35	92.1	31	93.9	25	96.2	18	100.0	
Normal	9	3.9	8	6.8	1	2.6	0	0.0	0	0.0	0	0.0	
Not tested	11	4.7	6	5.1	2	5.3	2	6.1	1	3.9	0	0.0	
X-Ray Cavity													0.144
No	150	70.4	66	63.5	30	85.7	22	71.0	18	72.0	14	77.8	
Yes	63	29.6	38	36.5	5	14.3	9	29.0	7	28.0	4	22.2	
Pulmonary													0.569
No	13	5.6	8	6.8	1	2.6	3	9.1	1	3.9	0	0.0	
Yes	220	94.4	110	93.2	37	97.4	30	90.9	25	96.2	18	100	

Definition of abbreviations: *SD* Standard Deviation, *IQR* Interquartile Range

^a^Pearson’s chi-square test

There was no significant differences between the patients within the 5 large GENType clusters in sputum smear or culture positivity (*p* > 0.05, Table 2). Abnormal chest radiography (*p* = 0.518), radiographic confirmation of cavitation (*p* = 0.144) or having previously had TB (*p* = 0.706, Table 2) were also not significantly different between the 5 GENTypes (Table 2). No multi-drug resistant (MDR) or extensively drug resistant (XDR) isolates were identified among the cases clustered in the 5 GENTypes analyzed. Six of the 233 cases were resistant to a single drug (two resistant to Isoniazid and four resistant to either Rifampin, Rifabutin, Amaikacin or Ofloxacin). All the resistant isolates had GENType G00010 (data not shown).

### Temporal distribution

**Fig. 1:**
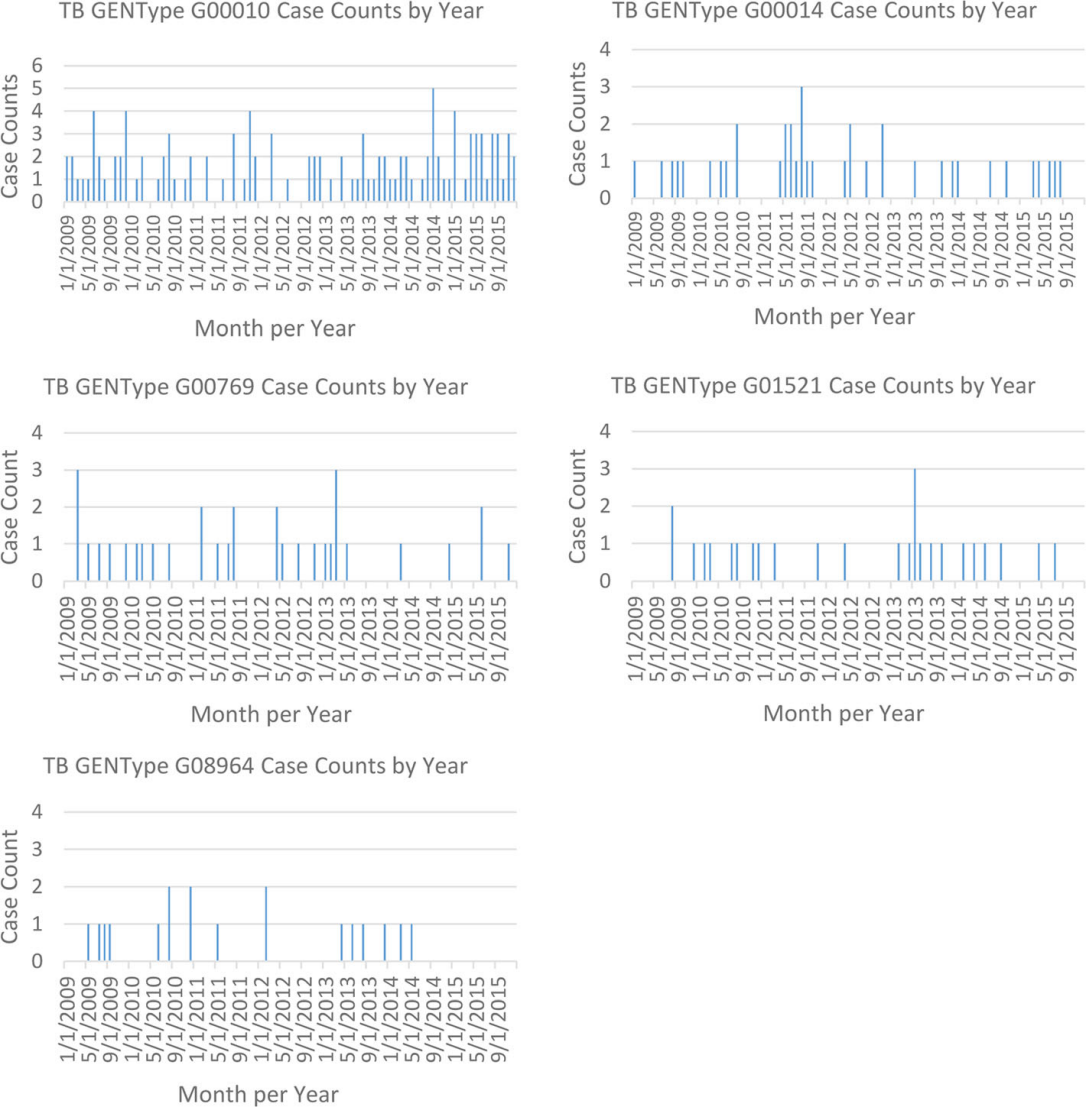
Epidemiologic curves for each cluster from January 2009 through December 2015

The epidemiologic curves show the preponderance of GENTypes G00010, G00014, G00769 and G01529 (Fig. 1). The GENType G008964 arose and circulated from midway through 2009 through the first half of 2014 in HC indicating that it was potentially an outbreak strain in HC that may have been eliminated in 2014 (Fig. 1).

### Spatial distribution of clusters

**Fig. 2:**
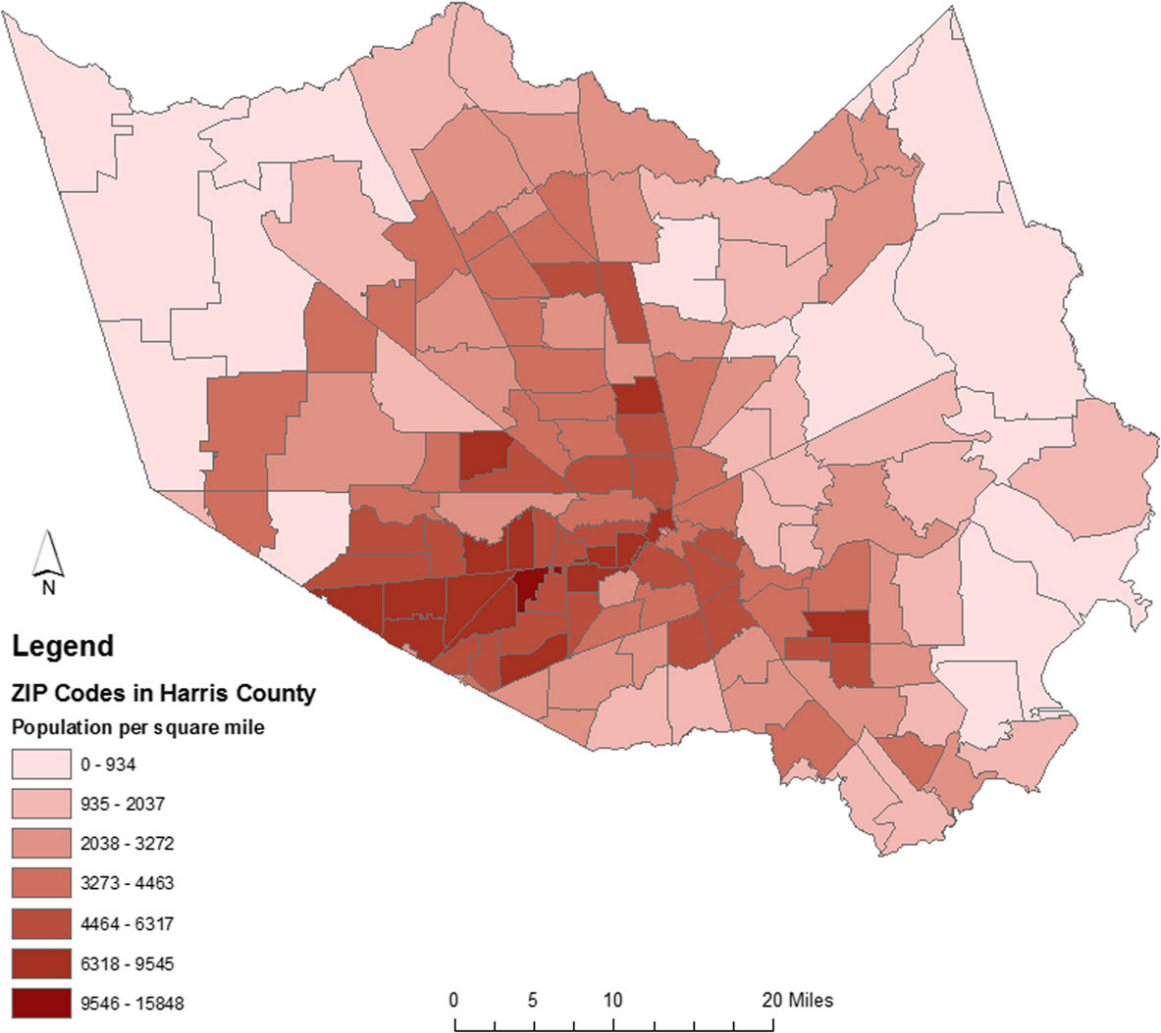
Choropleth Map showing population density of zip codes with Harris County based on 2010 U.S. Census data. Estimated population of each zip code is divided by the area of each zip code in square miles

**Fig. 3:**
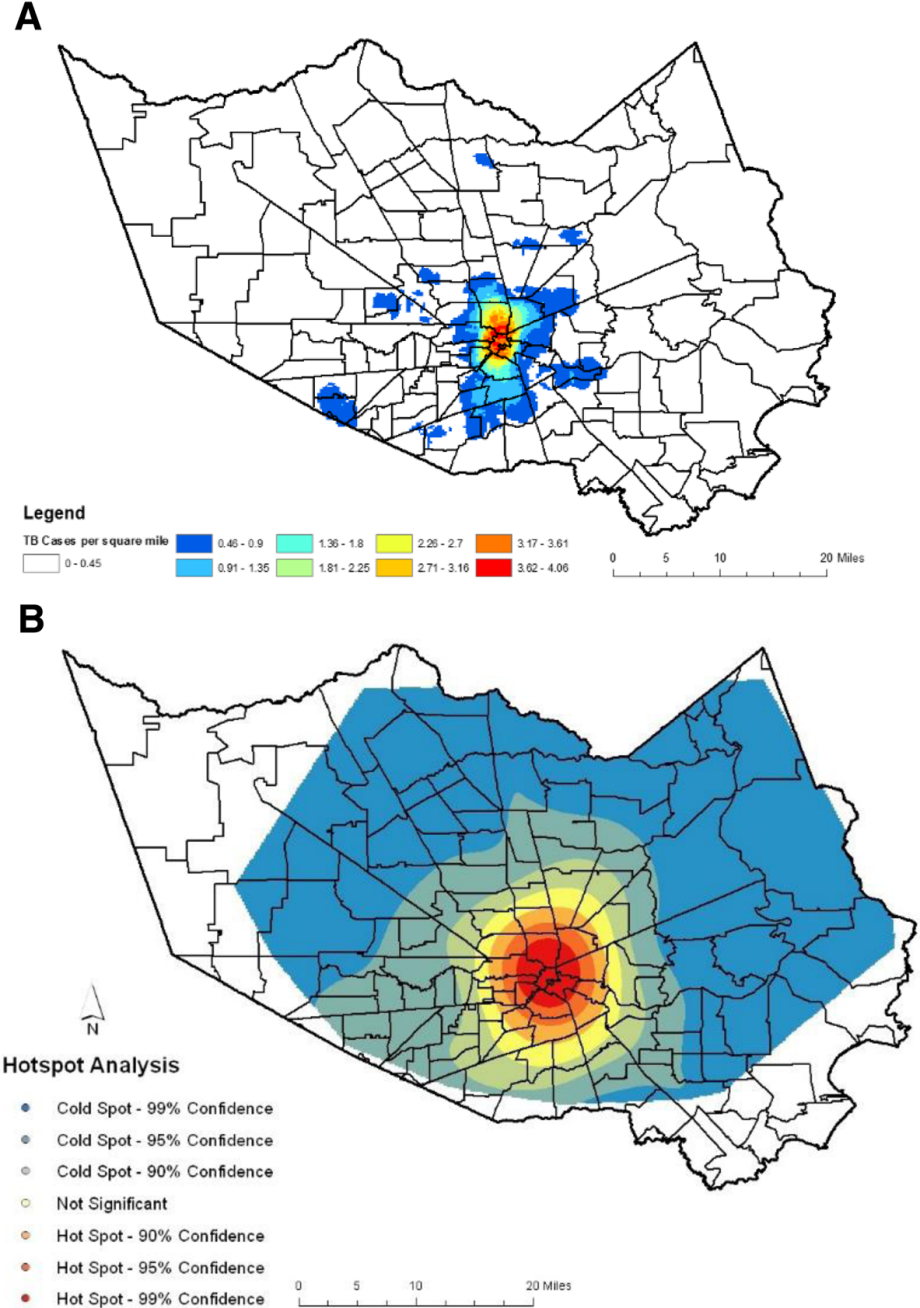
Maps of TB Cases in 5 largest clusters in Harris County, TX 2009–2015. **a**. Point Density Analysis. The number of TB cases in each zip code was divided by the area in square miles of each zip code. **b**. Hot spot Analysis Map shows the distribution of hot and cold spots of TB cases with the 5 most common GENTypes. In Hot spot Analysis z-scores and *p*-values are used to identify features with either significantly high amount of clustering (hot spot) or low amount of clustering (cold spots) spatially

**Fig. 4:**
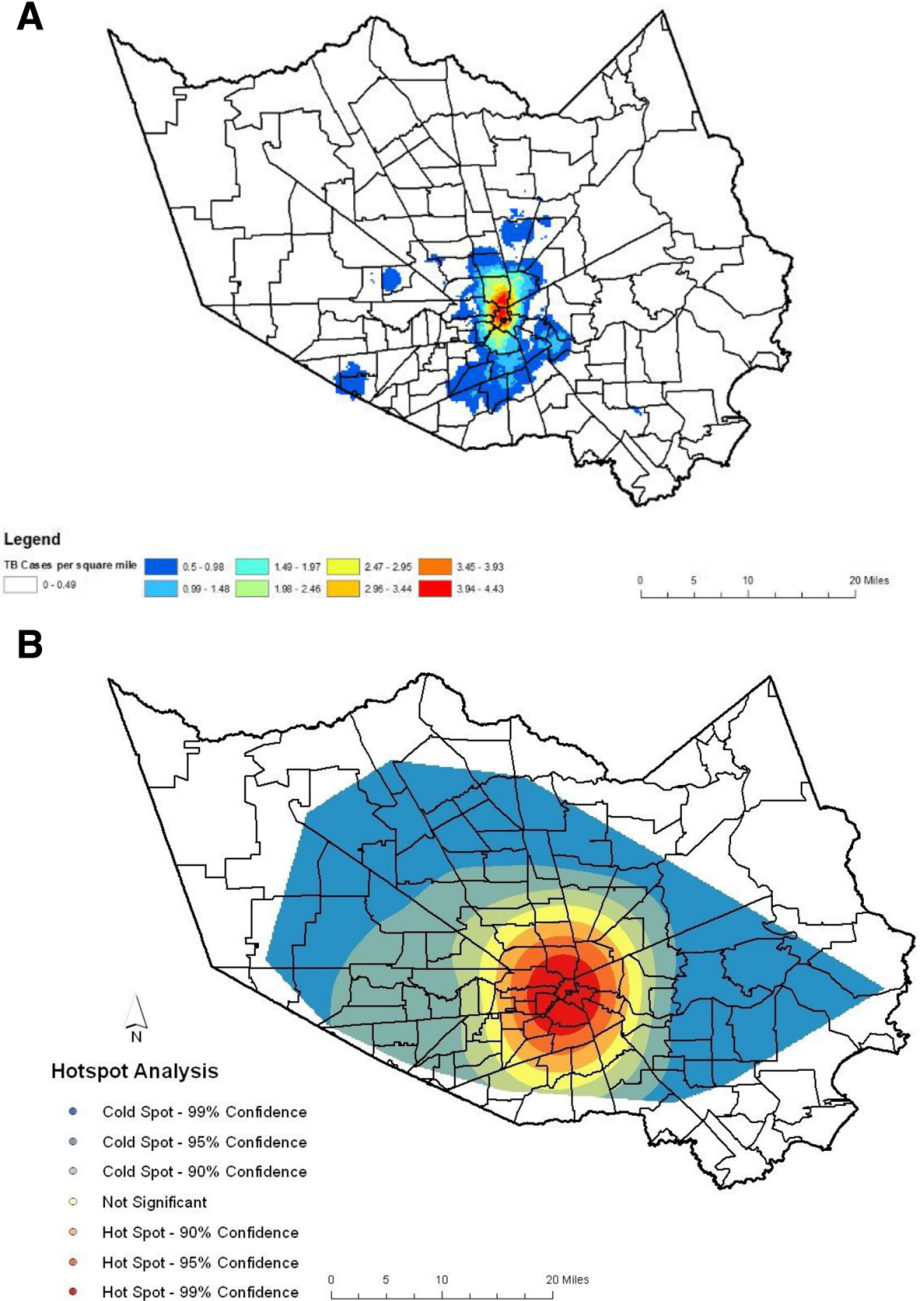
Map of TB Cases with GENType: G00010 in Harris County, TX 2009–2015. **a**. Point Density Analysis. The number of TB cases with GENType G00010 in each zip code was divided by the area in square miles of each zip code. **b**. Hot spot Analysis Map shows the distribution of hot and cold spots of TB cases with the GENType G00010. In Hot spot Analysis z-scores and *p*-values are used to identify features with either significantly high amount of clustering (hot spot) or low amount of clustering (cold spots) spatially

One hundred thirty-nine (139) zip codes in HC had genotyped TB cases between 2009 and 2015, and the 5 most common GENTypes were disseminated across 79 zip codes in HC. Population density is greater in the central regions of the county (Fig. 2), and the distribution of cases within the county reflects this trend. The kernel density surface analysis indicates that the Houston downtown area had a greater than average number of TB cases that fell within the GENType clusters of interest, and this is supported by hot spot analysis (Fig. 3a and b). The 118 cases of GENType G00010 appear to make up a large portion of the Houston inner city hot spot area (Fig. 4a and b), while a large concentration of cases other than GENType G00010 were clustered downtown and south and west of the downtown region.

## Discussion

In HC between 2009 and 2015, 1058 distinct GENTypes were identified, including 5 large *Mtb* clusters. The large clusters were seen primarily in those individuals born in the U.S. despite the large foreign-born population residing in HC (59% foreign born, 1325/2251) [1]. This finding is a departure from the national trends that show a higher proportion of reported TB cases among the foreign-born (66.4%) compared to the U.S.-born (33.6%) populations in 2015 [2]. TB cases occurring more often in U.S.-born than foreign-born in HC has been previously reported [8]. Clustering has also been seen more often in U.S.-born TB cases than foreign-born cases (36.0% versus 14.4%) in the U.S. [2]. The high rate of clustering in HC compared to the U.S., may be partially due to the dynamics of disease transmission occurring in the U.S.-born subpopulation, while TB cases in the foreign-born are reactivation of previous infection or acquired and imported while abroad. There is also a remote possibility that *Mtb* strains outside of the U.S. may be identical to endemic *Mtb* strains in HC. None of the large clusters identified in this study had any *Mtb* isolates identified as being MDR or XDR indicating that MDR/XDR TB is not likely being transmitted in HC although MDR/XDR TB infection cannot be determined and ruled out.

The most common GENType in HC during this study was G00010. This finding reflects national data as this genotype has been found to be one of the most common *Mtb* genotypes among genotyped TB cases in the United States from 2010 to 2015 [2, 9–11]. This *Mtb* strain is one of the oldest characterized strains in Houston, first reported in HC in 1995 [12]. Thirteen percent (13.3%) of TB cases identified as belonging in the most common clusters in HC occurred in in the foreign-born, and 45% (14/31) of the *Mtb* isolates in the foreign-born were GENType G00010. The lack of clustering among the foreign-born indicates that most individuals do not acquire TB in the U.S., but they acquire the *Mtb* infection abroad or from visitors from foreign countries. The few foreign-born TB cases in the large clusters were long term residents (median 18 years ±13.2 years) of the U.S. before they developed TB with a strain identified as highly clustered in HC indicating that they most likely acquired the disease in the U.S.

Three of the 5 largest genotype clusters during the study time frame had the Beijing spoligotype “000000000003771” [7] which has a reported global distribution [7]. A study conducted among 27 TB outbreaks in the U.S. between 2002 and 2008 found that 3 outbreaks involved Beijing family strains [13]. Another study conducted in Houston found that 25% of *Mtb* isolates from resident TB patients had a spoligotype pattern common to the Beijing family [14]. In addition, there is mounting evidence that this *Mtb* strain family is hypervirulent in animal models [15] which may help explain how 3 genotypes with the “000000000003771” spoligotype have become prevalent and possibly endemic in HC. From our data several prevalent strains appear to be transmitted among the U.S.-born males of black or Hispanic race/ethnicity.

Four of the five analyzed clusters in this study have been previously characterized in HC [3]. Teeter et al. described the 4 largest spoligotype/12 loci VNTR-MIRU defined clusters in HC from 2006 to 2012. The epidemiology of the described clusters appears similar in both time frames. GENType G01521, corresponds to Teeter and coworkers’ cluster D, which was described as “declining” after an outbreak in 2007–2008 [3], but G01521 has persisted in HC through 2015 indicating that the strain may have become endemic. The CDC defines the term endemic as “the constant presence and/or usual prevalence of a disease in a population within a geographic area” [16]. The CDC definition lacks a time component; however, 4 of the 5 strains in the study have been actively transmitted in the community for the 7 year duration of this study. To the authors, this indicates that these particular strains may be endemic within HC.

Hot spot analysis revealed that the largest geospatial location of TB cases was in the City of Houston inner city area. Of the TB cases analyzed, 16.3% were in homeless individuals of which 81.7% (31/38) had GENType G00010. The majority of the homeless individuals have been identified as living in the downtown area of Houston [17]. That is also the location of the largest concentration of homeless shelters and food pantries. Individuals diagnosed while incarcerated at the Harris County jail were assigned a residential zip code of 77,002, so these TB cases do not have a zip code that reflects a “true” residence, but a general location in which temporary housing occurred. Houston itself is a widespread metropolitan area covering 600 mile^2^ and characterized by urban sprawl. High density residential areas are generally found within the Interstate 610 Loop, and lower density suburbs are found outside the loop. A gradual shift has been seen in the population density to the southwest area of Houston over the last 30 years [18]. Houston’s Asian demographic is concentrated in the Braeswood to Bellaire sector found in the southwest region of Houston [19]. The endemic nature of the strains circulating in the inner city of Houston indicates that current TB control program strategies are inefficient or ineffective in the identification and treatment of contacts or exposed subpopulations that are highly mobile and may not be able or willing to identify contacts by name for follow-up investigations. This lends credence to the need and use of location-base, GIS and outbreak investigative surveillance methods as part of TB control strategies. A study by Feske et al. showed that persons living within specific census tracks in HC between 1995 and 2004 had a risk for TB disease equal to that of individuals living in a high burden country [20].

Clustering is used as a surrogate for recent transmission, but this surrogate can be prone to errors in characterizing TB transmission. Genotyping coverage increased in the U.S. from 86.9% in 2009 to 96.1% in 2015, and individual states had TB case genotype surveillance coverage ranging from 74.9% to 100% between 2013 and 2015 [2]. In Texas >95% of culture positive TB cases had *Mtb* isolates sent to the CDC for genotyping between 2011 and 2015 [2], and in HC, 95.5% of culture positive TB cases were genotyped in the analyzed time frame [1]. TB cases not having *Mtb* isolates sent for genotyping can lead to underestimation of transmission within a community [21], as well as, missed opportunities to identify individuals with unknown epidemiologic-links. States and jurisdictions with lower surveillance coverage will reduce the proportion of clustered cases in the U.S. This may partially account for the higher proportion of clustering seen among Texas and HC genotyped TB cases. Another issue that must be considered is the jurisdictional basis of the cluster definition. Clusters might be missed if the source case of a cluster lives in a different jurisdiction than the secondary case. This can be remedied by communication between TB programs in adjacent jurisdictions.

A limitation of this study is that the analyses does not contain all TB cases identified in HC in the time frame of interest. Not all of HC tuberculosis cases had *Mtb* isolates sent for genotyping, so our analysis is restricted to TB cases that had an *Mtb* isolate that was genotyped in the selected time interval. Omission of these cases could bias our studies if there was a selection bias in TB cases that were sent for molecular characterization. However, in Texas most of the culture positive TB cases (> 95%) had isolates sent for genotyping between 2011 and 2015 [2] making it unlikely that selection bias occurred. *Mtb* genotyping is subject to laboratory and reporting errors that can lead to false-positive culture results, and the discriminatory power of the genotyping techniques used may be insufficient to resolve different endemic strains being transmitted. This *Mtb* characterization methodology might lead to several clusters being grouped as a single cluster confusing outbreak/cluster investigations. Another limitation is in the geospatial data. This data set had zip code level data, so all geospatial analysis were limited to that level. Homeless individual’s residential zip code was based on the location where these individuals congregated at homeless shelters, soup kitchens or bunk houses, and individuals incarcerated at the time of diagnosis were given the zip code 77002. These zip codes for these groups may not reflect the “true” location where transmission occurred. Hot spot analysis were based on randomly placed points with the TB cases zip code rather than patients’ physical addresses introducing a degree of imprecision. However, despite this limitation the analysis still allows for policy and planning of resource allocation to public health jurisdictional areas of Houston and HC.

## Conclusions

Five large clusters with distinct GENTypes were found in HC between 2009 and 2015, and the most common GENType was G00010. These clusters occurred primarily among U.S. born individuals indicating that active transmission of *Mtb* was occurring in Harris County, Texas. Prevention of transmission is imperative in any TB control program, but control and prevention strategies also need a clear picture of the geospatial, temporal distribution and epidemiology of TB cases to be able to make informed decisions on resource allocation. Identifying and understanding the trends associated with subpopulations and neighborhoods with a high density of TB cases allows for targeted intervention strategies to be designed and implemented. Studying large *Mtb* clusters in Harris County has been prudent in our assessment of the ramifications of potentially transmitted endemic strains of *Mtb*. TB control programs with universal genotyping of TB cases should be able to monitor their ability to control and prevent TB disease in their jurisdiction by observing the decline or increase in the proportion of clustered TB cases over time and by evaluating *Mtb* genotyping trends with epidemiologic data during the same time periods. To see a significant drop in TB in Harris County we strongly recommend that health department TB programs re-evaluate their current contact identification strategies in the inner City of Houston area based on the 7 year trends described in this analysis and begin extensive implementation of additional control measures such a location-based surveillance, GIS utilization, and cluster investigations.

## Abbreviations

CDC: Centers for Disease Control and Prevention
GIS: Geographical Information System.
HC: Harris County.
*Mtb*: *Mycobacterium tuberculosis.*
RVCT: Report of Verified Case of Tuberculosis.
TB: Tuberculosis.
TX: Texas.
U.S.: United States
VNTR-MIRU: Variable number of tandem repeat – mycobacterial intersperse repetitive units
